# Supplementary material for: Novel Driver Strength Index highlights important cancer genes in TCGA PanCanAtlas patients
Source: PeerJ. 2022 Aug 11;10:e13860. doi: 10.7717/peerj.13860 (PMC9375969; doi:10.7717/peerj.13860)
Supplement: Supplemental Information 2 [file peerj-10-13860-s002.zip › COHORTS/genes plots/cohorts/2021_8_16_14_9_distribution_cohorts_females_PANCAN.pdf]

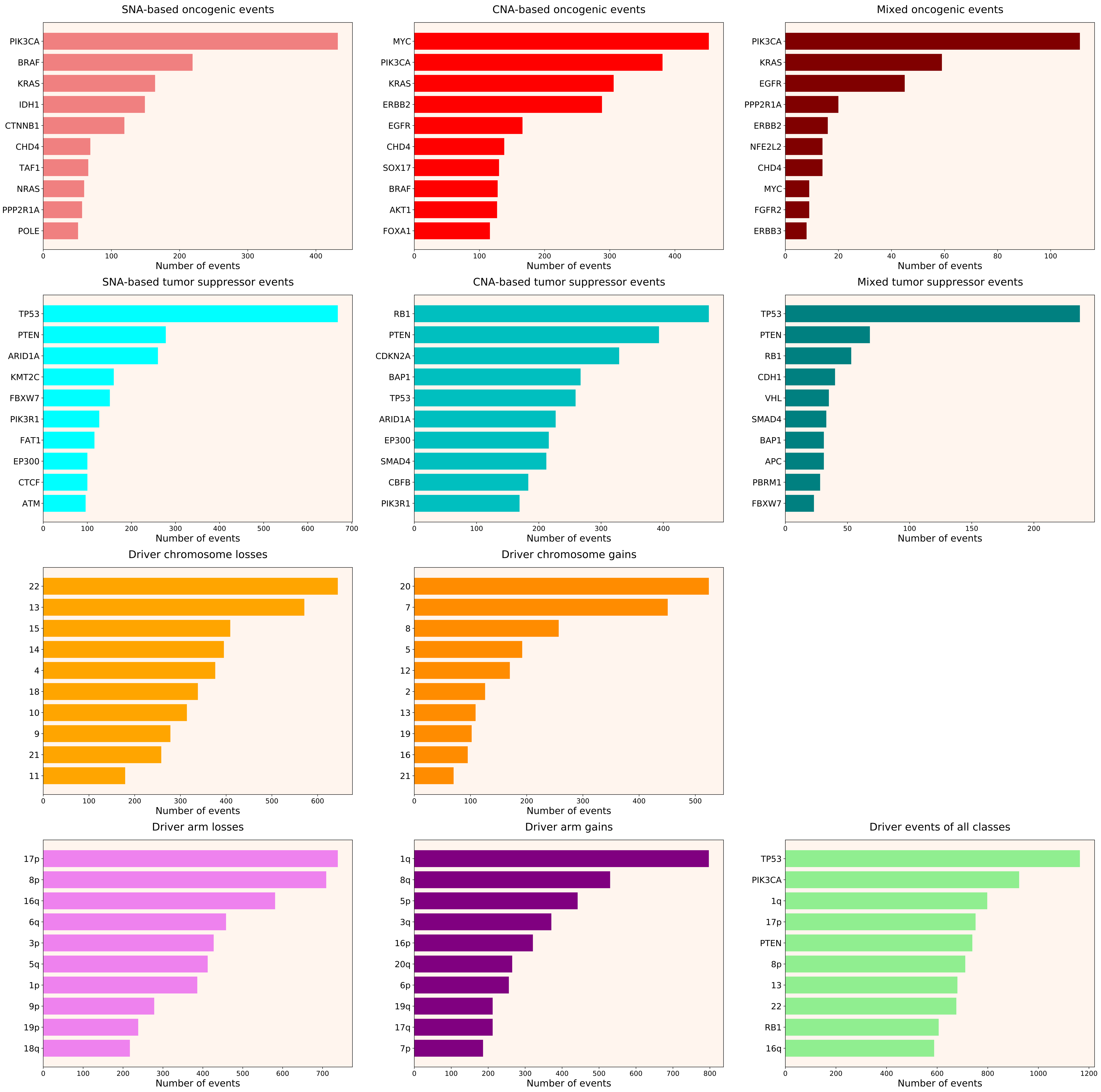

SNA-based oncogenic events
PIK3CA
BRAF
KRAS
IDH1
CTNNB1
CHD4
TAF1
NRAS
PPP2R1A
POLE
0
100
200
300
400
Number of events
CNA-based oncogenic events
MYC
PIK3CA
KRAS
ERBB2
EGFR
CHD4
SOX17
BRAF
AKT1
FOXA1
0
100
200
300
400
Number of events
Mixed oncogenic events
PIK3CA
KRAS
EGFR
PPP2R1A
ERBB2
NFE2L2
CHD4
MYC
FGFR2
ERBB3
0
20
40
60
80
100
Number of events
SNA-based tumor suppressor events
TP53
PTEN
ARID1A
KMT2C
FBXW7
PIK3R1
FAT1
EP300
CTCF
ATM
0
100
200
300
400
500
600
700
Number of events
CNA-based tumor suppressor events
RB1
PTEN
CDKN2A
BAP1
TP53
ARID1A
EP300
SMAD4
CBFB
PIK3R1
0
100
200
300
400
Number of events
Mixed tumor suppressor events
TP53
PTEN
RB1
CDH1
VHL
SMAD4
BAP1
APC
PBRM1
FBXW7
0
50
100
150
200
Number of events
Driver chromosome losses
22
13
15
14
4
18
10
9
21
11
0
100
200
300
400
500
600
Number of events
Driver chromosome gains
20
7
8
5
12
2
13
19
16
21
0
100
200
300
400
500
Number of events
Driver arm losses
17p
8p
16q
6q
3p
5q
1p
9p
19p
18q
0
100
200
300
400
500
600
700
Number of events
Driver arm gains
1q
8q
5p
3q
16p
20q
6p
19q
17q
7p
0
100
200
300
400
500
600
700
800
Number of events
Driver events of all classes
TP53
PIK3CA
1q
17p
PTEN
8p
13
22
RB1
16q
0
200
400
600
800
1000
1200
Number of events
